# Leukotrienes as Modifiers of Preclinical Atherosclerosis?

**DOI:** 10.1100/2012/490968

**Published:** 2012-05-01

**Authors:** Graziano Riccioni, Magnus Bäck

**Affiliations:** ^1^Cardiology Unit, San Camillo de Lellis Hospital, Manfredonia, Foggia, Italy; ^2^Department of Cardiology and Center for Molecular Medicine, Karolinska University Hospital, Stockholm, Sweden

## Abstract

Preclinical atherosclerosis represents a crucial period associated with several pathophysiological reactions in the vascular wall. Failure to diagnose preclinical atherosclerosis at this stage misses a major opportunity to prevent the long-term consequences of this disease. Surrogate biological and structural vascular markers are available to determine the presence and the extension of preclinical vascular injury in the general population. Examples of surrogate markers are carotid intima media thickness and biomarkers including high-sensitivity C-reactive protein, cell adhesion molecules and matrix metalloproteinases, and leukotrienes. Recently, leukotrienes have been implicated as mediators, biomarkers, and possible therapeutic targets in the context of subclinical atherosclerosis. The aim of this short paper is to focus on the relation between preclinical atherosclerosis and leukotrienes, with particular attention to the recent development on the use of leukotriene modifiers in the treatment of atherosclerosis.

## 1. Introduction

Atherosclerosis, a progressive disease of large arteries with a long asymptomatic phase, accounts for much of the global epidemic of cardiovascular disease (CVD). During the past decades, the concept of atherosclerosis has become refined with a focus on the inflammatory nature of the atherosclerotic lesion [1].

Inflammation in atherosclerosis involves alteration of the endothelial monolayer, which in the normal state resist prolonged contact with leukocytes. Risk factors such as elevated levels of low-density lipoprotein cholesterol (LDL-C) lead to endothelial changes [2]. The altered endothelium expresses a series of adhesion molecules, such as vascular cell adhesion molecule1 (VCAM-1) and P-selectin, which participate in the initiation of atherosclerosis [3]. A number of chemoattractants in addition drive the migration of adhered leukocytes into the arterial intima where the atherosclerotic lesions form. This migration result from the action of chemoattractants, such as monocyte chemoattractant protein 1 (MCP-1) and leukotriene B_4_. Atherosclerotic disease progression can lead eventually to acute cardiovascular events, such as acute myocardial infarction (AMI), unstable angina (UA) pectoris, sudden cardiac death, or stroke [4]. While the disease is still in a subclinical stage, however, the presence of atherosclerosis can be identified by several methods, including coronary angiography, intravascular ultrasonography (IVUS), B-mode ultrasonography, computed tomography (CT) scan, and magnetic resonance imaging (MRI). In addition, vascular biomarkers such as high-sensitivity C-reactive protein (hs-CRP) and cell adhesion molecules have proven to be useful to predict subclinical atherosclerosis. Recently, leukotrienes (LTs) have been implicated as mediators, biomarkers, and possible therapeutic targets in the context of subclinical atherosclerosis.

## 2. LTs: Definition, Synthesis, and Function

LTs are arachidonic acid (AA) derived lipid mediators of inflammation. The initial step in the formation of LTs is catalyzed by 5-lipoxygenase (5-LOX) in conjunction with its five lipoxygenase activating protein (FLAP) [5, 6]. Subsequently, LTC_4_ synthase leads to the formation of the family of cysteinyl leukotrienes (CysLTs) including leukotriene C_4_ (LTC_4_), leukotriene D_4_ (LTD_4_), and leukotriene E_4_ (LTE_4_), whereas the noncysteine-containing dihydroxyleukotriene B_4_ (LTB_4_) is formed through the action of the enzyme LTA_4_ hydrolase [6]. LTs are mainly produced by macrophages infiltrating atherosclerotic lesions and act in an autocrine/paracrine manner within the vascular wall. For example, LTB_4_ is a potent chemoattractant for monocytes, neutrophil granulocytes, and T lymphocytes, that promotes leukocyte adhesion to vascular endothelium, augments vascular permeability, and promotes vascular smooth cells (VSMCs) proliferation and migration [7, 8]. The two receptors for LTB_4_ are termed BLT_1_ and BLT_2_. The BLT_1_ receptor is the high-affinity receptor specific for LTB_4_ expressed in leukocytes, vascular smooth muscle cells, and endothelial cells and mediates chemotaxis [9]; BLT_2_ is a pharmacologically distinct receptor ubiquitously expressed and displays low affinity for LTB_4_ and also binds other agonists [10]. For example, the thromboxane synthase metabolite 12-L-hydroxy-5,8,10-heptadecatrienoic acid (12-HHT) has been identified as a more potent ligand for BLT_2_ than LTB_4_, although the specific role of 12-HHT activation of leukotriene receptors in CVD has not been extensively explored [11].

CysLTs (LTC_4_, LTD_4_, and LTE_4_) are potent vasoconstrictors and also enhance vascular permeability [12]. In addition, *in vivo* studies have shown that CysLTs, reduce coronary blood flow, decrease myocardial contractility, and regulate blood pressure [13]. CysLTs also stimulate proliferation of arterial smooth muscle cells and promote P-selectin surface expression, von Willebrand factor secretion, and platelet-activating factor (PAF) synthesis in cultured endothelial cells (EC) [14]. CysLTs exert their biological effects by activating specific receptors termed CysLT_1_ and CysLT_2_, of which the CysLT_1_ receptor is blocked by the antileukotrienes used clinically in the treatment of asthma [10].

## 3. Endothelial Dysfunction: A Preclinical Atherosclerosis Phase

Endothelial injury may be one starting point for atherosclerosis. Such injury can result from a variety of factors, including increased local shear forces from hypertension, elevated plasma concentrations of LDL-C, chemical toxins in for example cigarette smoke, and diabetes mellitus. These factors decrease (EC) production of nitric oxide (NO), thereby impairing vasodilatory capacity, normal barrier, and protective functions of the vascular endothelium. Dysfunctional EC in addition upregulate, a number of adhesion molecules, which promote the binding of circulating monocytes to vascular EC [15]. Whereas EC in nonatherosclerotic arteries are devoid of BLT receptors, the endothelium lining human carotid atherosclerotic lesions have been demonstrated to be positive for the BLT_1_ receptor [16]. In line with these findings, human umbilical vein endothelial cells (HUVECs) exhibit BLT_1_ upregulation after stimulation with the proinflammatory cytokines LPS and IL-1*β* in vitro [17]. In addition, *in vitro* studies have demonstrated a LTB_4_-induced release of vasoactive factors via BLT receptor activation [18, 19]. These reports provide evidence for a role of LTB_4_ in regulating endothelial function.

The CysLT_2_ receptor appears to be the dominating CysLT receptor in normal EC [20], but endothelial CysLT_1_ receptor expression can be induced after prolonged exposure to IL-1*β* [21]. Furthermore, studies of human brain tissue have revealed CysLT_1_ receptor immunostaining in microvascular EC [22]. Studies in isolated vessels have associated CysLT_1_ receptor signaling with the release of contractile factors, whereas the CysLT_2_ receptor appears preferentially coupled to the release of NO [23]. Furthermore, LTC_4_ and LTD_4_ upregulate endothelial P-selectin expression [24] and increase the transcription of the CXC chemokines MIP2-*α* [25] and IL-8 [26].

## 4. Immunoactivation: A Pivotal Step in Atherosclerosis Progression

The dysfunctional endothelium will hence promote the recruitment of immune cells. Once attached, monocytes will be exposed to several chemokines that promote the transmigration of bound monocytes into the subendothelial space, and to colony stimulating factors, that promotes the differentiation of monocytes into macrophages. In addition to the stimulation of endothelial adhesion molecules discussed above, the direct chemotactic activity of LTB_4_ may also participate in monocyte/macrophage accumulation during atherogenesis.

Macrophages expressing scavenger receptors (SRA, CD-36) will bind and promote the internalization of oxidized LDL (LDL_ox_) and a broad range of other particles and cell fragments [15]. As the macrophages progressively accumulate more and more cholesterol, cytosolic lipid droplets form, and the macrophage become a lipid-laden foam cell. Although the exact role of LTs in oxidized LDL uptake remains to be established, the enhanced CD-36 expression and increased uptake of LDL_ox_ induced by 4-hydroxynonenal are blunted when 5-LO activity is inhibited by a pharmacological inhibitor, and in macrophages derived from 5-LO-deficient mice [27]. Furthermore, LDL_ox_ upregulates FLAP expression in monocytic cell lines [28], suggesting also an enhancing function of LDL_ox_ on leukotriene-driven inflammation in atherosclerosis. In addition to monocytes, T-lymphocytes infiltrate the developing lesion site from both the intimal and adventitial aspects of the vessel wall [29, 30]. LTB_4_ is a potent chemoattractant for T-lymphocytes and may also be a key mediator in a lymphocyte-monocyte cross-talk enhancing inflammatory circuits in atherosclerosis [31]. In addition, LTB_4_ stimulates the release of matrix metalloproteinases (MMPs) from T-lymphocytes in vitro [32]. The MMP family of enzymes is involved in arterial wall extracellular matrix degradation and remodeling [33]. In addition to stimulating T-lymphocyte MMPs, LTs also induce MMP release from neutrophil granulocytes [34], monocytes [35], and vascular smooth muscle cells [36, 37].

## 5. Biomarkers of Subclinical Atherosclerosis

Dysfunction of the endothelium may be considered as an early and potentially reversible step in the process of atherogenesis, and numerous methods have been developed to assess endothelial status and large artery stiffness [38]. Several biochemical markers have been identified, which correlate with coronary artery disease (CAD) and conventional CVD risk factors [39]. Such biomarkers include, LDL_ox_, hs-CRP, endothelial progenitor cells (EPC), prothrombotic factors such as von Willebrand factor (VWF), and inflammatory markers including tumor necrosis factor-*α* (TNF-*α*), IL-6, and intracellular adhesion molecule-1 (ICAM-1) [40, 41], as well as different members of the MMP family [33].

## 6. LTs and Subclinical Atherosclerosis: Genetic Evidence

The first suggestion for the involvement of the LTs pathway in subclinical atherosclerosis was provided by Dwyer and coworkers, reporting an association of the number of Sp1/Egr1 motifs within the 5-LO promoter sequence with an increased carotid intima-media thickness [42]. In the latter study, carriers of the variant genotypes in addition exhibited 2-fold higher levels of hs-CRP. Genetic variations in other constituents of the LTs pathway have also been demonstrated to correlate with measures of subclinical atherosclerosis. For example, a SNP within the LTC_4_ synthase promoter correlated with carotid intima-media thickness and coronary calcium [43]. Finally, the genetic association of the LTs pathway with atherosclerosis is also supported by studies of CVD outcomes, in which variations in the genes encoding FLAP, LTC_4_ synthase, and LTA_4_ hydrolase [44] have been associated with a higher prevalence of MI and/or stroke.

## 7. LTs and Atherosclerosis: Experimental Evidence

Targeting the 5-LO enzyme in animal models of atherosclerosis have generated contradictory results [45, 46]. However, pharmacological inhibition of FLAP in different atherosclerosis prone mice has shown reduced lesion size [31, 47, 48].

LTB_4_ is one of the most potent chemoattractants formed within the atherosclerotic lesion, and several leukocyte subpopulations with importance for atherosclerosis development have been shown to be activated by LTB_4_ [49]. In human atherosclerotic lesions, macrophages stain positive for both BLT receptor subtypes [16], and *in vitro* studies have supported a role for both the high-affinity BLT_1_ receptor and the low-affinity BLT_2_ receptor in macrophage recruitment to atherosclerotic lesions [16, 34]. Furthermore, BLT receptor expression on T-lymphocytes may play a key role in the immunological reactions within the atherosclerotic lesion [31]. In addition to macrophages and T-lymphocytes, also granulocytes express BLT receptors [49] and, although incompletely explored in atherosclerosis, LTB_4_ was recently demonstrated as a key mediator of neutrophil chemotaxis and activation in human aortic abdominal aneurysms [34].

In addition to leukocyte activation, LTB_4_ also induces effects on structural components of the vascular wall [16, 36, 50, 51]. In addition to endothelium-dependent vasomotor responses discussed above, LTB_4_ also induces contractions of some vascular segments lacking a functional endothelium [52], and BLT receptor expression was subsequently demonstrated on VSMC [16, 53]. Furthermore, experimental models targeting the BLT_1_ receptor have indicated the importance of LTB_4_-induced VSMC migration and proliferation in the development of atherosclerosis [16, 53]. Finally, BLT receptor antagonism reduces the intimal hyperplasia in response to balloon angioplasty with stent implantation in hypercholesterolemic rabbits [36], suggesting a potential use of antileukotrienes in the prevention of restenosis following coronary percutaneous interventions. Likewise, either pharmacological [54, 55] or genetic [55, 56] targeting of the BLT receptor reduces atherosclerotic lesion size in hyperlipidemic mice.

CysLTs induce contraction of atherosclerotic human coronary arteries [57]. Recently, the CysLT pathway was associated with aortic stenosis, a vascular inflammation which shares several characteristics with atherosclerosis [58]. In this context, LTC_4_ stimulation of valvular myofibroblasts increased reactive oxygen species production and induced calcification. In addition, the local expression levels of 5LO correlated with the severity of valvular disease, suggesting that the role of the leukotriene pathway in CVD may extend beyond atherosclerosis.

Pharmacological targeting of the CysLT_1_ receptor retards atherosclerotic lesion growth in hyperlipidemic mice [59, 60] and reduces the intimal hyperplasia in response to vascular injury [61].

## 8. LTs as Biomarkers of Atherosclerosis and CV Risk

*Ex vivo* incubations of human atherosclerotic lesions have shown an increased release of both CysLTs [62] and LTB4 [63] compared with healthy human vessels. Brezinski et al. [64] detected an increased local LTs production during coronary interventions, and a systemic increase in LTB_4_ formation has been detected through *ex vivo* stimulation of leukocytes derived from patients with a history of AMI [65]. The latter approach was recently used in a study of subjects with obstructive sleep apnea [66], which demonstrated correlations of LTB_4_ concentrations with measures of carotid artery remodelling. Patients with obstructive sleep apnoea in addition exhibit urinary excretion of LTE_4_, which is associated with the severity of sleep apnoea and obesity [67]. Since both sleep apnoea and obesity are established CVD risk factors, the latter findings warrant further investigation of the potential of LTs as biomarkers of atherosclerosis and CVD risk. Finally, oral concentrations of LTs may represent another interesting approach for the evaluation of LTs as CVD biomarkers [68]. For example, subjects with high concentrations of CysLTs in gingival crevicular fluid have an increased carotid artery wall thickness, regardless of their dental status [69].

## 9. LT Modifiers and Atherosclerosis: The Clinical Experience

Inflammation plays an important role in atherosclerosis process. In particular, many enzymes associated with the 5LO pathway are abundantly expressed in arterial walls of patients afflicted with various lesion stages of atherosclerosis of the aorta and of coronary arteries [41]. In a randomized, placebo-controlled trial, Hakonarson et al. [70] demonstrated that an inhibitor of FLAP (DG-031) led to significant and dose-dependent suppression of plasmatic biomarkers that are associated with increased risk of AMI events. The authors randomized 191 patients, who carry at-risk variants in the arachidonate 5-lipoxygenase-activating protein (ALOX5AP) gene (87%) or in the leukotriene A4 hydrolase gene (13%), to receive 250 mg/d of DG-031, 500 mg/d of DG-031, 750 mg/d of DG-031, or placebo for 4 weeks. In patients with specific at-risk variants of 2 genes in the leukotriene pathway, DG-031 led to significant and dose-dependent suppression of hs-CRP that is associated with increased risk of AMI events. Even Allayee et al. [71] provided evidence that montelukast and low-dose theophylline decrease certain inflammatory and lipid CVD risk factors. In this study, the authors randomized 133 patients with moderate-to-severe asthma to receive either montelukast (10 mg/d), theophylline (300 mg/d), or placebo for 6 months. Asthmatic patients receiving montelukast and, to some extent, low-dose theophylline have lower levels of CVD-associated inflammatory biomarkers (hs-CRP, IL-6) and lipid levels.

In acute coronary syndrome (ACS), a potent 5LO inhibitor (VIA-2291) reduces leukotriene production as demonstrated by Tardif et al. [72] in a double blind trial with placebo. In this study, 191 patients were randomly assigned 3 weeks after an ACS to receive 25, 50, or 100 mg VIA-2291 or placebo daily for 12 weeks. A significant reduction of urine leukotriene LTE_4_ was obtained in all dose groups with the absence of serious adverse events. In this study, a subset of patients (*n* = 60) underwent a 64-slice coronary CT examination at baseline continued on study medication for a total of 24 weeks and underwent a repeat scan. A significant reduction in noncalcified plaque volume at 24 weeks versus placebo was observed in VIA-2291—treated groups in the 34 of these 60 patients in whom this end point was analyzable.

In a registry-based nation-wide Swedish cohort of almost 7 million subjects followed for 3.5 years, montelukast use was associated with a borderline significant protective effect on recurrent stroke [73]. Based on significant interactions, a subgroup analysis revealed that montelukast use was associated with a significantly reduced risk of recurrent stroke in subjects not receiving angiotensin-modifying drugs. In the same study, a stratified analysis revealed that montelukast use significantly reduced the risk of recurrent myocardial infarction in males [73]. These results provide a first indication for beneficial effects of the CysLT1 receptor antagonist montelukast in the secondary prevention of stroke and myocardial infarction.

## 10. Conclusions

The inflammatory process of atherosclerosis is associated with several pathophysiological reactions characterized by the production of LTs, which induce proinflammatory signaling through activation of specific BLT and CysLT receptors. CysLTs and LTB_4_ exert a range of the proinflammatory effects and have proved to be important mediators in inflammatory conditions such as preclinical atherosclerosis. For this reason, leukotriene synthesis inhibitors and leukotriene receptor antagonists have been suggested to induce beneficial effects at preclinical stages of the atherosclerotic process.

Further experimental and clinical studies are needed to determine the potential therapeutic strategies targeting the LT pathway in CVD for to increase a potential use of these drugs in cardiovascular and cerebrovascular diseases [74, 75]. The exact role of LTRAs in disease management is still evolving. Large-scale, controlled trials are needed to determine the effectiveness and the safety deriving from the use of LTRAs in CVD.
